# Pulmonary drug delivery of quercetin through scalable PEGylated mixed micelles of Gelucire® and Tetronic®

**DOI:** 10.1039/d5ra09376g

**Published:** 2026-02-17

**Authors:** Deep Bhalani, Sadafara A. Pillai, Aakash Shukla, Gitika Kharkwal, Debes Ray, Vinod K. Aswal, Pratap Bahadur

**Affiliations:** a School of Sciences, P P Savani University NH-8, GETCO, Near Biltech, Kosamba Surat-394125 Gujarat India sa.pillai@ppsu.ac.in; b Division of Biological Sciences, ICMR-National Institute of Occupational Health Ahmedabad-380016 Gujarat India; c Solid State Physics Division, Bhabha Atomic Research Centre (BARC) Mumbai-400085 Maharashtra India; d Department of Chemistry, Veer Narmad South Gujarat University (VNSGU) Udhana-Magdalla Road Surat 395007 Gujarat India

## Abstract

Many potent anticancer agents suffer from poor water solubility, limiting their industrial translation into effective formulations. Although single micellar systems have been explored to address this issue, they often exhibit low stability and encapsulation efficiency. To overcome these limitations, this study developed and characterized PEG-based mixed micelles composed of Gelucire® 48/16 and Tetronic® 1304 (T1304), aiming to improve the solubilization and cytotoxicity of a model lung cancer drug, quercetin (QCT). The micelles were extensively analysed using cloud point (CP), small-angle neutron scattering (SANS), and high-performance liquid chromatography (HPLC) techniques. SANS confirmed that Gelucire® 48/16 formed spherical or ellipsoidal micelles depending on composition with T1304. Micellar growth and improved drug encapsulation are noticed in saline conditions through the salting-out effect. *In vitro* cytotoxicity studies in lung epithelial adenocarcinoma (A549) cells demonstrated that Gelucire® 48/16 micelles enhanced the cytotoxic effect of QCT, while T1304 provided controlled release, with the mixed system offering intermediate modulation. The results indicate that mixed micellar systems showed a potential cytotoxic effect *via* increased ROS generation and DNA damage, ultimately damaging cancer cells. Additionally, our findings support a practical and commercially viable approach for enhancing the solubility and therapeutic efficacy of hydrophobic drugs, which can be further useful in various biomedical applications, such as healthcare formulations and drug delivery.

## Abstract 2

Mechanistic illustration of composition- and salt-driven micellar evolution in Gelucire®-T1304 self-assembled nanostructures.

## Graphical abstract

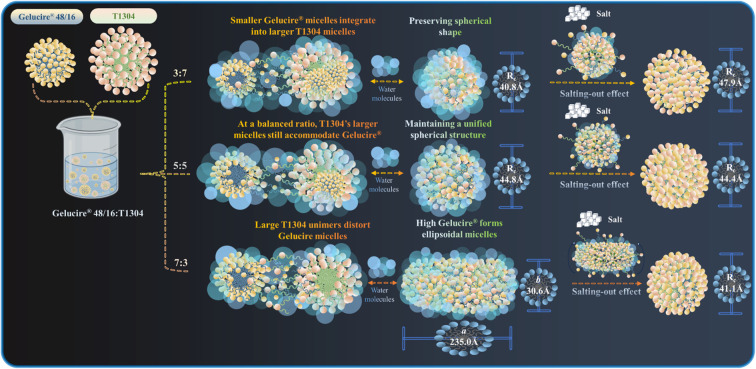

## 1. Introduction

Most people around the world who smoke increase their risk of developing cancer, especially lung cancer, which remains the second most prevalent cause of cancer-related deaths worldwide.^1,2^ Although survival rates for many types of cancer have improved, progress in lung cancer treatment has remained slow, mainly because of its late detection, when chemotherapy, surgical removal, and radiation therapy are the only available options.^3^ However, these treatments often lack specificity and can cause significant damage to the surrounding healthy tissues.^4,5^ Further, drug solubility and low bioavailability continue to be major problems in the healthcare industry. In the past decade, nanotechnology has gained attention as a promising strategy for overcoming these limitations in treating cancers because of its distinct advantages.^6,7^ Therapeutic drug delivery through nanocarrier platforms has demonstrated promising potential to enhance the solubility and bioavailability of different hydrophobic anticancer drugs by selectively targeting cancer cells while reducing damage to surrounding healthy tissue.^8–10^

Polymeric micelles are a class of nanocarriers widely utilized in anticancer drug delivery systems because they can encapsulate hydrophobic drugs and enhance their solubility, stability, and bioavailability.^11,12^ Tetronic® represents a distinctive class of pH-responsive and thermoresponsive block copolymeric surfactants, consisting of four polyethylene glycol–polypropylene glycol (PEG–PPG) arms linked with a central ethylenediamine group. This unique architecture and the availability of wide hydrophilic–lipophilic balance (HLB) values (due to varying PEO/PPO ratios) impart excellent biocompatibility and solubilization of drugs, making these surfactants particularly valuable as nanovectors in delivery systems.^13^ In addition to possessing the capacity to solubilize drugs in various environments, these copolymer molecules are very sensitive to ionic strength and show a considerable response to different types of additives.^14^ The central diamine component within the Tetronic® copolymer is responsible for its very high responsiveness to pH variations in Tetronic® copolymers.^15,16^ This characteristic is particularly relevant to cancer treatment since cancer tissues tend to have higher acidity than normal tissues.^17^ Therefore, the Tetronic® copolymer becomes protonated in acidic environments, which generates charged ammonium ions and repels one another, disrupting the formation of micelles and allowing drug release. In contrast, in higher pH environments, the amine groups remain relatively neutral, facilitating the formation of micelles and the encapsulation of drugs by Tetronic® copolymers.^14,18^ Through this mechanism, it is possible to provide targeted delivery of therapies to tumor tissues while being stabilized in normal tissues, thereby limiting the cytotoxic effect of therapies on healthy tissues surrounding cancer tissues.^19^ Thus, Tetronic® copolymers provide a better way of delivering a pH-responsive drug compared to their linear analogue Pluronics®, which do not respond to pH.^14^ Extensive studies on Tetronic® systems have deepened our understanding of their micellar behavior and drug solubilization under varying pH conditions, further highlighting their potential in targeted cancer therapies. Lorenzo *et al.*^13^ demonstrated the solubilization of griseofulvin in T904 and further investigated how pH conditions modulate this process. Complementing these findings, Kadam *et al.*^20^ demonstrated the impact of multiple variables, such as temperature, pH and concentration, as well as salt concentration, on the T904 micelles, showing that the T904 micelles grow larger when all these factors are combined under a basic pH, along with higher temperatures and higher salt concentrations. Vyas *et al.*^21^ performed research to test the solubility of two anti-cancer substances, quercetin (QCT) and curcumin when performed using the temperature and pH-dependent method of self-assembly using different Tetronic® (T304, T904, and T908) copolymers, with similar results. However, single-component micellar systems often suffer from poor dilution stability, uncontrolled release kinetics, and reduced encapsulation efficiency, limiting their application in healthcare industries.^22^ In order to overcome these problems, one of the strategies researched is the formation of mixed micellar systems by mixing various amphiphiles. Mixed micelles provide a way to enhance stability, optimize solubilization, control release kinetics, and enable the tuning of physicochemical characteristics to improve therapeutic efficacy. Several researchers have explored the potential of Tetronic® copolymers to enhance the solubility and stability of poorly water-soluble drugs, particularly through their ability to form mixed micelles.^23–25^ For instance, Pillai *et al.*^26^ utilized a mixed micellar system with T904 and T901 to increase the aqueous solubility of curcumin and significantly improve its solubilization. Similarly, Cagel *et al.*^27^ formulated mixed micelles using T1107 and d-α-tocopheryl polyethylene glycol succinate (TPGS) to deliver doxorubicin, showing pH-sensitive drug release and increased cytotoxicity. Ribeiro and researcher^28^ reported that single and mixed micelles with Tetronic® T904, T908, T1107, and T1307 enhanced both the solubility and stability of the antiglaucoma drug ethoxzolamide, making them promising nanocarriers for sustained therapy.

Gelucire® 48/16 is an important class of low molecular weight PEG ester-based non-ionic surfactants and notably exhibits excellent solubilizing capacity for poorly water-soluble drugs,^29^ such as solid dispersions,^30–37^ nanoparticles,^38^ self-emulsifying drug delivery systems (SEDDS),^39^ and mixed micelles^40^ owing to its ability to enhance drug solubility due to its high HLB of 16.^41^ Though there are several reports on different physical forms of Gelucire®-based formulations, very few are available in aqueous media. Jadhav *et al.*^42^ investigated the micellar behavior of Gelucire® 44/14 using small-angle neutron scattering, finding spherical structures at low temperatures and a shift to cylindrical or rod-like shapes near the CMC and at higher temperatures. Date *et al.*^43^ studied Gelucire® 50/13 as a stabilizer for solid lipid nanoparticles and nanostructured lipid carriers and observed that it enabled the nanosizing of solid lipids and improved the stability of formulations. Similarly, Antunes *et al.*^44^ highlighted the potential of Gelucire® 44/14 in enhancing drug performance by showing significant improvements in the solubility and dissolution rate of carbamazepine. Extending these applications further, Alshawwa *et al.*^41^ utilized electrospraying to fabricate Celecoxib stabilized with Gelucire® 48/16, successfully enhancing solubility and oral bioavailability. There are also reports on other mixed micellar systems that have been developed with Gelucire® and other excipients to enhance drug delivery performance.^45–49^ Patil *et al.*^49^ formulated mixed micelles using Gelucire® 44/14 and Pluronic® F127 for curcumin, achieving improved micellar stability, encapsulation efficiency, and solubilization compared to single polymeric micelles. These mixed micelles enhanced the solubility of curcumin and significantly improved its cytotoxicity against the human lung cancer cell A549. Similarly, Singh *et al.*^47^ developed mixed micelles for exemestane using a combination of Pluronic L121, F127, and Gelucire® 44/14. These mixed micellar systems enhanced drug solubility and bioavailability and improved *in vitro* cytotoxicity against MCF-7 cancer cells. In line with these findings, Shinde *et al.*^46^ observed that a binary mixture of Gelucire® 48/16 and TPGS exhibited synergistic behavior, resulting in efficient curcumin solubilization.

Understanding the importance of both the class of amphiphiles in anticancer drug delivery, *i.e.* enhanced solubilizing power of Gelucire® and pH-responsive nature of Tetronic® copolymers, in our previous study,^50^ we compared the efficiency of encapsulation between Tetronic® T1304 and Gelucire® 48/16. Interestingly, despite the general perception that polymeric micelles exhibit superior solubilizing capacity to low molecular weight nonionic surfactants, Gelucire® 48/16 demonstrated greater improvement in drug solubility than Tetronic® 1304. However, it is important to note that there is a significant difference between polymeric micelles and surfactant micelles from a physicochemical viewpoint. Polymeric units associate to form micelles at low concentrations, known as the critical aggregation concentration (CAC), which is much lower than the surfactant CMC values. Additionally, polymeric micelles offer better kinetic stability and preserve their structure for an extended period, whereas low molecular weight amphiphiles are less stable and break down easily under pharmaceutical conditions upon dilution due to their high CMC. Therefore, for better kinetic stability and lower toxicity, polymeric micelles are preferred as drug nanocarriers.^51^ It can be assumed that by mixing Gelucire® 48/16 with polymeric micelles of T1304, the solubilizing potential of the copolymer can be significantly improved, providing the dual advantages of increased solubility and stability of the lung cancer drug. Although mixed micelles have been widely studied for drug delivery, to the best of our knowledge, no study has specifically investigated the combination of Gelucire® 48/16 and T1304. The complete absence of research, despite its potential relevance to the healthcare industry, motivated us to explore this unique pairing in depth. The goal of our study was to develop mixed micelles formed by T1304 and Gelucire® 48/16 in order to increase the physicochemical properties, such as solubilization and stability, and ultimately increase the therapeutic potential of a model drug used to treat lung cancer. The behavior of these systems is characterized through analytical techniques, including cloud point (CP) measurements and small-angle neutron scattering (SANS). These results help elucidate the effects on micelle shape, size distribution, and phase behaviour. Additionally, this study examines the solubilization efficiency of mixed micellar systems for the anticancer effects of QCT in lung cancer, with and without salt, using high-performance liquid chromatography (HPLC). We also investigated the cytotoxic potential of QCT in water and micellar formulations using human lung epithelial adenocarcinoma (A549) cells to address the poor solubility and limited cellular uptake of QCT. The cytotoxic effects were evaluated using the MTT assay, which was supported by reactive oxygen species (ROS) generation and Hoechst 33342 nuclear staining to assess oxidative stress and nuclear morphology. This work presents a promising strategy for nanocarrier-based delivery to improve the therapeutic efficacy of anticancer drugs in lung cancer, which may find potential applications in the oncology healthcare industry.

## 2. Experimental

### 2.1. Chemicals used

**Fig. 1:**
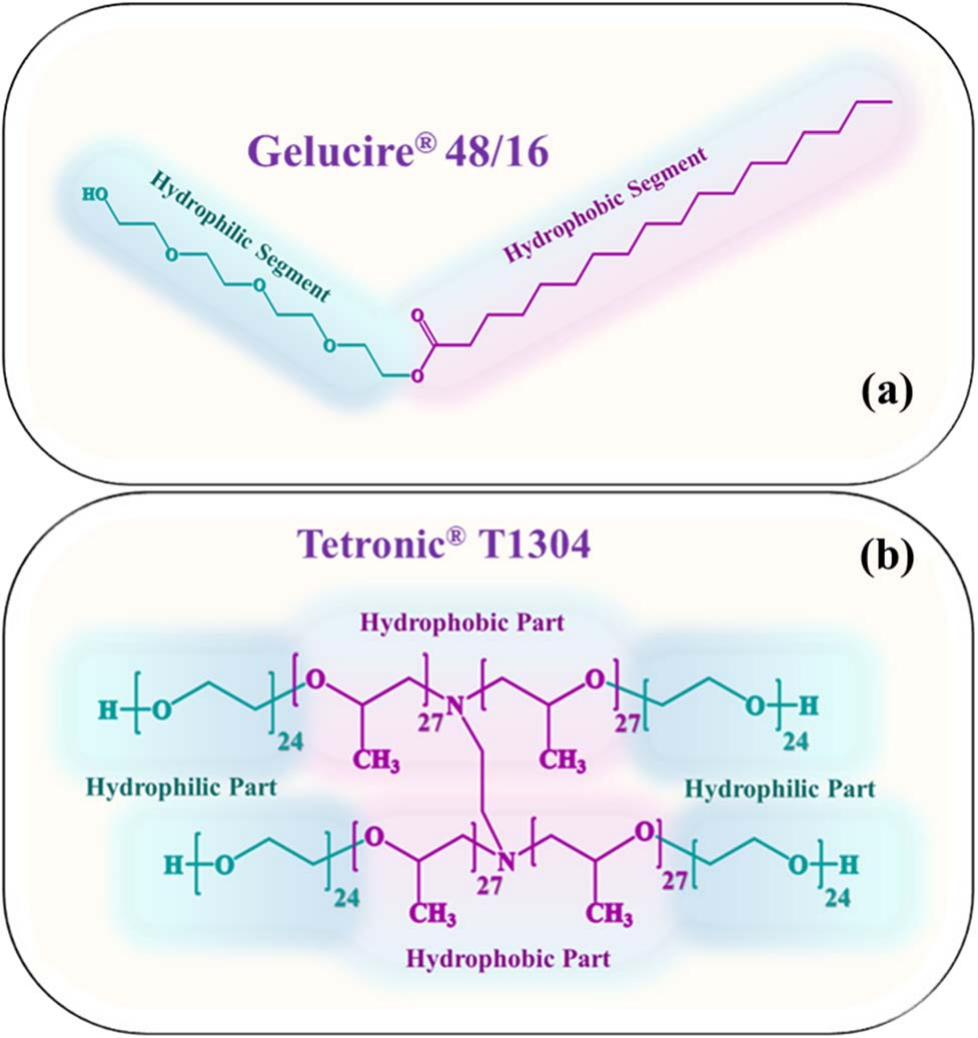
Chemical structure of (a) Gelucire® 48/16 and (b) Tetronic® T1304.

Gelucire® 48/16, a gift sample, was received from Gattefossé, France. Tetronic® T1304, a gift sample, was received from BASF Corp., India. The anticancer drug, quercetin, was purchased from TCI-JPN. No purification was performed before it was used. NaCl was a 98–99% pure analytical grade sample purchased from Sigma Aldrich (India) and was also used as received without any further purification. Deionized water from the Milipore Milli-Q system was used to prepare all the samples, except SANS. Moreover, 99.9 atom% pure D_2_O, purchased from Sigma Aldrich (India), was used for the sample preparation for SANS. The structure of the PEGylated surfactants is shown in Fig. 1.

### 2.2. Methods

#### 2.2.1. Preparation of stock solutions

Stock solutions of mixed micellar systems containing Gelucire® 48/16 and T1304 were prepared using three weight ratios: 30 : 70, 50 : 50, and 70 : 30 (Gelucire® 48/16:T1304). For each ratio, the amphiphiles were accurately weighed and dissolved in distilled water to obtain a final concentration of 1% w/v for the combined surfactant system. Each stock solution was prepared in a volume of 2 mL, in which the required amount of NaCl varied from 0 M to 2 M, as specified in the formulation codes listed below.

#### 2.2.2. Cloud point (CP) determination

The CP of all the solutions was measured in the presence and absence of varying concentrations of salt. The experiments were conducted in a temperature-controlled water bath, with the temperature increased at a constant heating rate of 1 °C min^−1^. A 2 mL solution was placed in a 5 mL glass vial, and the thermometer was inserted and stirred continuously to ensure a uniform temperature at which the solution first appeared turbid upon visual inspection. All the measured CP values were repeated three times for accuracy, with a deviation of up to ±1 °C.

#### 2.2.3. Small-angle neutron scattering (SANS)

SANS measurements were carried out using Gelucire® 48/16 and T1304 mixed micellar formulations in deuterated water (D_2_O), in both the presence and absence of salt. The experiments were conducted at the Dhruva reactor facility at Bhabha Atomic Research Centre (BARC), Mumbai, India. Samples were placed in ultraviolet-grade quartz cells with a 5 mm path length and sealed using Teflon stoppers to prevent evaporation or contamination. The sample-to-detector distance was set to 1.8 meters. The scattering vector (*Q*) was in the range of 0.017 < *Q* < 0.35 Å^−1^, where *Q* is defined using the following equation:*Q* = (4π/*λ*) × sin(*θ*/2),where *θ* is the scattering angle and *λ* is the wavelength of the incident neutrons.

All measurements were taken at a controlled temperature of 30 °C. The raw SANS data were processed by subtracting the background noise, accounting for the empty cell, correcting for sample transmission, and then converting them to absolute scattering cross-section units. The data were analysed using the SASFIT application.

The differential scattering cross-section per unit volume, d*Σ*/d*Ω*(*Q*), for micellar solutions is given byd*Σ*/d*Ω*(*Q*) = *nV*^2^(*ρ*_p_ − *ρ*_s_)^2^*P*(*Q*)*S*(*Q*) + *B*,where *n* is the number density of micelles, *V* is the volume of each micelle, and *ρ*_p_ and *ρ*_s_ are the scattering length densities of the micelles and solvent, respectively. *P*(*Q*) is the form factor describing the shape and size of individual micelles, *S*(*Q*) is the structure factor accounting for interactions between micelles, and *B* is a constant that represents incoherent background scattering, primarily from hydrogen atoms in the sample.

#### 2.2.4. High-performance liquid chromatography (HPLC)

##### 2.2.4.1. Drug solubilization technique

The solubilisation of drug QCT in all the above-described systems (shown in Table 1) was analysed using reverse phase HPLC (LC-2010, AHT; Shimadzu, Japan). For the separation in the HPLC chromatogram, an ODS C18 column (250 × 4.6 mm, 5 µm particle size) with a 100 Å pore size (Make: Thermo Scientific) was used.

**Table 1:** Composition of mixed micellar systems with varying Gelucire® 48/16:T1304 ratios and NaCl concentrations

Formulation code	Gelucire® 48/16 (%)	Tetronic® 1304 (%)	[NaCl], M
A1	30	70	0
A2	30	70	0.156
A3	30	70	0.5
A4	30	70	1.0
A5	30	70	1.5
A6	30	70	2.0
B1	50	50	0
B2	50	50	0.156
B3	50	50	0.5
B4	50	50	1.0
B5	50	50	1.5
B6	50	50	2.0
C1	70	30	0
C2	70	30	0.156
C3	70	30	0.5
C4	70	30	1.0
C5	70	30	1.5
C6	70	30	2.0

For the chromatographic system operation, to analyse and record the data, the LC Solution software was used. The mobile phase for the drug QCT used was water : acetonitrile : methanol (200 : 200 : 600 v/v). The samples were detected using a UV detector at a wavelength of 262 nm for QCT. Sample injection volumes were 20 µL with a mobile phase flow rate of 1.0 mL min^−1^, and the total run time was kept at 10 minutes.

Each micellar and mixed-micellar formulation was prepared in three separate batches (*n* = 3) and injected in triplicate per batch (*n* = 9) to ensure analytical repeatability.

##### 2.2.4.2. Drug loading amount and encapsulation efficiency by HPLC

To determine the drug loading amount and encapsulation efficiency of the surfactant and micellar mixture of the polymer, we prepared all the drug-loaded pure and mixed micellar solutions with varying concentrations of NaCl and a standard solution with the drug to compare the data. For the standard solution, an accurate 5 mg of the drug was dissolved in a pure solvent in which the drug was completely soluble. For the other drug-loaded micellar solution, take a 1% w/v of all the pure and mixed micellar copolymeric solution, and the salt was added in different concentrations. For the saturated drug-loaded solution, 5 mg of QCT was added to all the pure and mixed micellar-salt systems. The solutions were sonicated at room temperature for 90 min in an ultrasonic bath and kept overnight to equilibrate. The solutions were centrifuged at 10 000 rpm for 10 min and passed through a 0.22 µm nylon filter to remove undissolved particles and contamination. This step was not intended to separate free drug from micelle-associated drug but rather to ensure that subsequent analyses were performed on particle-free solutions. All the samples were analysed by using the HPLC method. All the measurements were performed at 30 °C.

The drug loading amount (DLA) and encapsulation efficiency (%EE) were calculated from the HPLC analysis area percentage results:
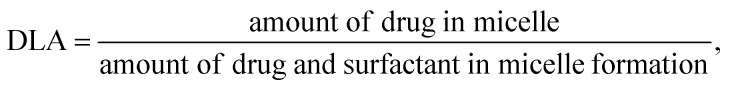

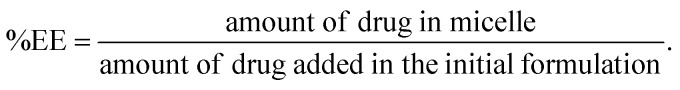


#### 2.2.5. *In vitro* cell viability assay

The 3-(4, 5-dimethylthiazol-2-yl)2, 5-diphenyltetrazolium bromide (MTT) assay assesses the cell viability of the anticancer drug QCT, both independently and in combination with carriers such as 1% w/v Gelucire® 48/16, 1% w/v T1304, and B1 formulation. The tetrazolium salt MTT was employed to examine mitochondrial function. The mitochondrial succinate dehydrogenase enzyme catalyzed the enzymatic reduction of MTT by converting the tetrazolium ring into an insoluble purple formazan.^52^ The cytotoxicity of QCT was evaluated by initially seeding A549 cells at a density of 2 × 10^5^ cells per mL (100 µL per well) into 96-well plates, followed by the addition of QCT at final concentrations of 100 µg mL^−1^, 50 µg mL^−1^, 25 µg mL^−1^, 12.5 µg mL^−1^, and 6.25 µg mL^−1^, all in quadruplicate. Untreated cells functioned as a positive control for viability and QCT in a cell-free culture medium, serving as an internal control for potential background interference. The plates were incubated for 24 hours under controlled conditions. Each well was administered 10 µL of MTT solution (0.5 mg mL^−1^), following a 24-hour incubation. Following the incubation period (four hours at 37 °C, 5% CO_2_), formazan crystals were dissolved in dimethyl sulfoxide (DMSO), and the optical density was measured at a reference wavelength of 570 nm using a multimode analyzer (Biotek, Synergy H1).

The following formula is used to determine the cell viability ratio:
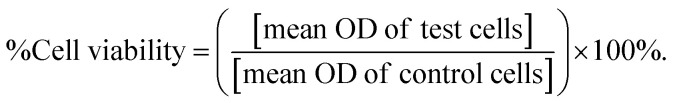


The concentration response was used to calculate the IC_50_ value, which was then expressed in µg mL^−1^.

#### 2.2.6. DCF-DA assay

Intracellular reactive oxygen species (ROS) generation was assessed using 2′,7′-dichlorofluorescein-diacetate (DCF-DA), as previously described in the literature.^53^ A549 cells (1 × 10^4^ cells per well) were seeded into a 96-well flat, bottom, and black plate and treated with various concentrations (6.25 µg mL^−1^, 12.5 µg mL^−1^, and 25 µg mL^−1^) of QCT in micelle-free aqueous media, Gelucire® 48/16, T1304, and Gelucire® 48/16 and T1304 mixed micelles for 24 hours. Following treatment, 10 µM DCF-DA was added to each well, and the cells were incubated at 37 °C for 30 minutes in the dark. Fluorescence intensity was then measured using a fluorescence spectrophotometer (Synergy-H1, BioTek). The results were expressed as the percentage increase in fluorescence intensity relative to the untreated control cells. Fluorescence microscopy images were also captured for both untreated cells and those treated with the IC_50_ concentration of each formulation.

#### 2.2.7. Hoechst 33342 nuclear staining assay

Hoechst nuclear staining was performed following the protocol described in the literature.^54^ A549 cells (3 × 10^5^ cells) were seeded into a 6-well plate and treated with the IC_50_ concentrations of QCT in micelle-free aqueous media, Gelucire® 48/16 and T1304 micelles, and B1 formulation for 24 hours. Subsequently, the cells were stained with Hoechst 33342 solution (0.5 µg mL^−1^) for 5–10 minutes at room temperature in the dark. Nuclear morphology was then examined using a fluorescence microscope.

### 2.3. Statistical analysis

The values presented correspond to the means of the measured observations. The results of cytotoxicity and ROS detection were presented as the mean ± SE (standard error) by performing one-way analysis of variance (ANOVA-SPSS 10.0, SPSS Inc., Chicago, IL) and Dunnett's (2-sided) post hoc test (Origin Pro 2021, Origin Lab, Northampton, MA, USA). A *p*-value < 0.05 was considered statistically significant.

## 3. Results and discussion

### 3.1. Cloud point (CP) measurements

Clouding of nonionic amphiphiles is proportional to their solubility in water, which decreases as the system temperature reaches the CP.^55,56^ This has been correlated with changes in the hydrophilic PEG segment. It is often identified by the dehydration of the hydrophilic PEG moiety at higher solution temperatures due to the breaking up of hydrogen bonds between water and PEG moieties of the copolymer, with the progressive increase in the temperature of PEG-based nonionic amphiphiles. Above the cloud point, the system separates into two isotropic phases. The phases appear to consist of an almost micelle-free dilute solution of these amphiphiles at a concentration equal to its CMC at that temperature, and a surfactant-rich phase that occurs only when the solution is above its cloud point.^57^ Depletion of water renders the transparent dispersion turbid, as a result of which it undergoes separation into water- and surfactant-rich phases. The phase separation is reversible, and upon cooling, the two separated phases merge to form a clear solution once again. In this context, demonstrating not only thermal stability but also robustness under processing and storage conditions is a key requirement for large-scale manufacturing in the healthcare industry.

This study examines the CP of binary mixtures in a total concentration of 1% w/v (as shown in Table 1).

**Fig. 2:**
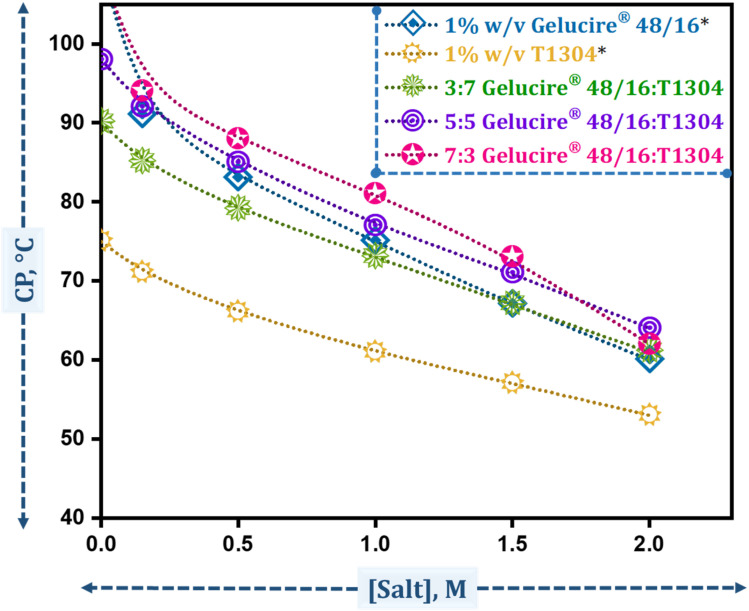
Variation in the CP of 1% w/v Gelucire® 48/16 and 1% w/v T1304 and their mixed micelles in the presence of different concentrations of salt. [*Data taken from Bhalani *et al.*^50^].

Gelucire® 48/16 exhibits a CP above 100 °C, while T1304 shows a CP at ∼76 °C, as reported in our previous study.^50^ In their formulations, A1, B1, and C1 show CP of 90 °C, 97 °C, and >100 °C, respectively (as shown in Fig. 2). These high cloud points indicate that micellar systems are thermally stable and do not undergo phase separation near physiological temperatures.^58^ Although the cloud point of these amphiphiles can be easily modified by adding an additive, it is important to investigate the influence of salt, commonly used as a pharmaceutical excipient, on these micellar systems.^59^ We added NaCl at various concentrations, as outlined in Table 1, to assess the effect of salt on all formulations and noted a significant decrease in the cloud point (CP) values. At saline concentrations, all formulations showed only a slight change in CP, confirming their applicability since the clouding temperatures remained considerably higher than physiological temperatures. Even at salt concentrations as high as 2 M, the CP remained above 60 °C, an improvement compared to the pure solutions in the referenced document. This suggests that the mixed micellar systems outperform the pure ones. Several factors may contribute to this effect: (a) the addition of salt, particularly less polarizable chloride ion, may increase the polarity of water, (b) a general salting-out effect may occur that reduces the hydration of hydrophilic segments by decreasing the availability of water molecules for hydrogen bonding with polar groups,^55^ (c) salt may disrupt water–PEG hydrogen bonds,^60^ and (d) salt can extract water molecules from the core region, promoting phase separation at a lower temperature.^61^ These findings demonstrate that mixed micellar systems retain high thermal stability even in saline environments, making them promising candidates for drug delivery applications where exposure to physiological and elevated ionic conditions is inevitable.

### 3.2. Small-angle neutron scattering (SANS)

SANS is a crucial technique to scrutinize the exact constitution and characteristics of block copolymers and surfactants. These measurements were intended to provide insight into the various morphological changes in the systems. To determine the precise morphology and size of the different formulations detailed in Table 1, SANS measurements are performed at 30 °C.

**Fig. 3:**
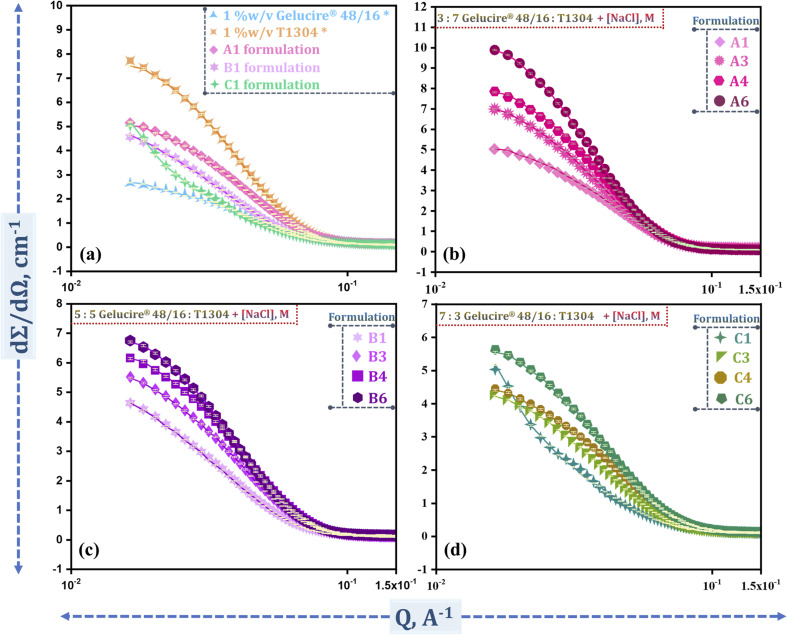
Scattering intensity profile for the (a) individual components and selected representative formulation without NaCl; (b) formulations with a 3 : 7 Gelucire® 48/16:T1304 weight ratio at varying NaCl molarities (A1, A3, A4, and A6); (c) formulations with a 5 : 5 Gelucire® 48/16:T1304 weight ratio at varying NaCl molarities (B1, B3, B4, and B6); and (d) formulations with a 7 : 3 Gelucire® 48/16:T1304 weight ratio at varying NaCl molarities (C1, C3, C4, and C6). [*Data taken from Bhalani *et al.*^50^].

Fig. 3(a) illustrates the morphology of these formulations. Fig. 3(b–d) depicts the scattering intensity profiles of these micellar systems in the presence of increasing NaCl concentrations. From the data, it is evident that the addition of NaCl influences micellar structure and size. Specifically, increasing salt concentration leads to enhanced scattering intensity, indicating micellar growth and changes in interaction among the micelles.^24^

As demonstrated in our previous study,^50^ both Gelucire® 48/16 and T1304 individually form spherical micelles. In the mixed micellar system composed of these PEG-based nonionic surfactants, the structural behavior of micelles is highly dependent on their ratio, which is influenced by the size and compatibility of the individual components. Upon mixing both surfactants in a 3 : 7 ratio of Gelucire® 48/16:T1304 (A1 formulation), the micellar system is predominantly governed by T1304. The smaller Gelucire® micelles smoothly integrate into the larger T1304 micellar structure, which provides sufficient space to accommodate the Gelucire® molecules without altering the overall spherical morphology with initial core radii of 40.8 Å, as illustrated in Table 2. In the presence of salt, this formulation consistently formed spherical micelles across all tested salt concentrations, with minor variations in core and polydispersity (PI). A similar integration effect is observed even at an equimolar ratio of 5 : 5 Gelucire® 48/16:T1304 (B1 formulation) with an *R*_c_ of 44.8 Å. This behavior is attributed to the inherently larger micellar size of T1304. T1304 micelles still accommodate Gelucire® although the assembly tends to get more disturbed in comparison to the A1 formulation. The relatively larger size of T1304 micelles allows them to maintain structural integrity and hold the Gelucire® without breaking apart. These formulations demonstrate stable spherical micelle formation under increasing salt concentrations with only subtle changes in size. This consistency in the core radius values across A1, A3, A4, A6 and B1, B3, B4, B6 formulations suggests that different morphologies do not exist simultaneously and that only spherical micelles with slight variations in sizes exist within the micellar systems. Importantly, despite variations in formulation components and in the presence of salt, the morphology remained unchanged; only spherical micelles were observed. This demonstrates that the addition of salt does not alter the micellar structure or lead to the formation of additional micellar species. Furthermore, the consistently low PI value supports the formation of uniform and nearly monodisperse micelles, reinforcing the conclusion that homogeneous species of spherical micelles predominate in these formulations. Interestingly, when the ratio is 7 : 3 in favour of Gelucire® (C1 formulation), it shows a distinct behaviour. In the absence of salt, this system formed ellipsoidal micelles, with a semi-major axis (*a* = 235.0 Å) and semi-minor axis (*b* = 30.6 Å), as determined by an ellipsoidal core–shell model (Table 2). This anisotropic morphology is likely due to the higher proportion of Gelucire® 48/16 in the formulation composition, which tries to penetrate the pre-existing T1304 micelles, leading to distortion in the spherical morphology. Although the ratio of Gelucire® 48/16 is higher than that of T1304, the individual T1304 monomers are significantly larger. Further, the higher concentration of Gelucire® 48/16 results in increased interaction pressure, leading to a distortion of the micellar structure. In this scenario, Gelucire® 48/16 attempts to penetrate the T1304 micelles, which leads to changes in shape and results in more stretched micelles. However, the influence of NaCl is particularly pronounced in systems with a higher Gelucire® 48/16 content. Gelucire® 48/16 is more hydrophilic than T1304, and in the presence of salt (a strong water structure-maker), the surrounding environment becomes more hydrophobic due to the disruption of water structure and reduced hydration of the polar head group. This diminished hydration promotes a transition from ellipsoidal to spherical micellar morphology, caused by micelles becoming more compact and stable. In the presence of a certain salt concentration, the micellar structure stabilizes in a spherical form, with minimal fluctuation in size or dispersity. The slight size reduction in formulations A4, B3, B4, and C4 is attributed to dehydration-induced compaction of the micellar corona, particularly of the PEG chains. However, at higher salt concentrations (2 M), both surfactants are similarly affected, resulting in a modest increase in micellar size due to chain dehydration.

**Table 2:** SANS measurement data for selected formulations (A1, A3, A4, A6; B1, B3, B4, B6; C1, C3, C4, and C6) of single and mixed micellar systems

Sr. no.	Formulation	Core radius *R*_c_ (Å)	Polydispersity	Micellar structure
1	1% w/v Gelucire® 48/16^a^	31.7	0.34	Spherical
2	1% w/v T1304^a^	43.6	0.23	Spherical
3	A1	40.8	0.25	Spherical
4	A3	45.1	0.24	Spherical
5	A4	43.7	0.23	Spherical
6	A6	47.9	0.22	Spherical
7	B1	44.8	0.28	Spherical
8	B3	41.8	0.26	Spherical
9	B4	40.7	0.25	Spherical
10	B6	44.4	0.23	Spherical
11	C1	Semi-major axis, *a* = 235.0	0.25	Ellipsoidal
		Semi-minor axis, *b* = 30.6		
12	C3	41.6	0.26	Spherical
13	C4	37.0	0.27	Spherical
14	C6	41.1	0.25	Spherical

a Data taken from Bhalani *et al.*^50^

This behavior reflects the dynamic nature of the micellar system, where there is a constant exchange of unimers between micelles. Not only does T1304 incorporate Gelucire® 48/16, but some T1304 unimers also exit from the micellar assembly. This adaptability highlights the complex interplay between composition, structure, and micellar behavior. Overall, the SANS data demonstrate a dynamic and competitive interplay between Gelucire® 48/16 and T1304 in mixed micelles, with NaCl playing a pivotal role in modulating micellar morphology and size. These findings highlight that precise control over micellar size and structure is crucial for enhancing drug solubilization, stability, and delivery efficiency in pharmaceutical and healthcare applications.

### 3.3. High-performance liquid chromatography (HPLC) study of encapsulation efficiency

**Fig. 4:**
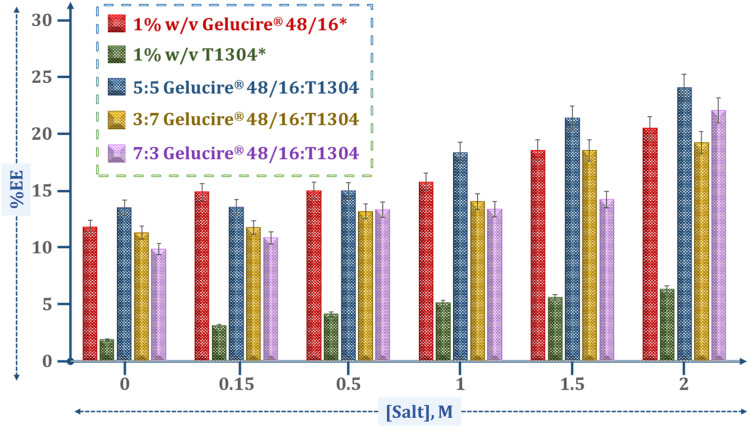
Encapsulation efficiency (%EE) of QCT in various formulations containing 1% w/v Gelucire® 48/16 (red), 1% w/v T1304 (green), and their mixture 5 : 5 Gelucire® 48/16:T1304 (blue), 3 : 7 Gelucire® 48/16:T1304 (yellow), and 7 : 3 Gelucire® 48/16:T1304 (purple) at different molar concentrations of salt (0–2) M.

The encapsulation efficiency (%EE) of all formulations for QCT is examined by HPLC, which is an accurate technique for quantitative analysis of small amounts of a drug entrapped in the micellar core. Our previous study^50^ showed that the solubilizing capacity of Gelucire® 48/16 is many times higher than T1304 due to the strong hydrophobic interactions between the fatty acid of Gelucire® 48/16 and drug molecules, which enhance the solubilization process, making it easier for QCT to be incorporated into the micellar core formed by Gelucire® 48/16. In this study, we observed that the %EE of all formulations mostly alternates between Gelucire® 48/16 and T1304. Fig. 4 shows the extent to which QCT is solubilized in the micellar solution as a function of salt concentration, clearly demonstrating that increasing salt concentration enhances QCT solubility. Many researchers have used Tetronic® copolymers to solubilize poorly water-soluble drugs, such as QCT and curcumin.^14,18,62^Table 3 illustrates the resulting data of the %EE of mixed micellar systems calculated from HPLC data, which shows that the solubility of the drug in every system increases as the concentration of salt increases.

**Table 3:** Encapsulation efficiency (%EE) and drug loading amount (DLA, mg) of QCT in various formulations (A1–C6)

Formulation	%EE	DLA mg
A1	11.29	0.56
A2	11.73	0.59
A3	13.15	0.66
A4	14.01	0.70
A5	18.51	0.93
A6	19.2	0.96
B1	13.48	0.67
B2	13.52	0.68
B3	14.95	0.75
B4	18.33	0.92
B5	21.35	1.07
B6	24.03	1.20
C1	9.84	0.49
C2	10.83	0.54
C3	13.31	0.67
C4	13.34	0.67
C5	14.20	0.71
C6	22.03	1.10

The data presented highlights the %EE and DLA (in mg) of formulations containing various ratios of Gelucire® 48/16 and T1304 in the presence of increasing concentrations of NaCl. For formulations A1, B1, and C1, the %EE and DLA ranged from 11.29%, 13.48%, and 9.84%, and from 0.56 mg, 0.67 mg, and 0.49 mg, respectively, showing moderate performance. As the NaCl concentration increased, all formulations improved in both %EE and DLA, with the most notable enhancement observed in the B1 formulation. Formulation B6 reached the highest encapsulation efficiency (24.03%) and drug loading amount (1.20 mg), suggesting a synergistic effect of the balanced excipient ratio and ionic strength on drug entrapment. Increasing NaCl concentration consistently enhanced the drug encapsulation performance across all formulations, with B6 showing the most pronounced benefit. The capacity of surfactants to solubilize the drug is closely linked to the available internal volume of the micelle, which is essential for accommodating the hydrophobic drug.^63^ Salt enhances drug solubility primarily by increasing the drug's interaction with polar solvents, like water.^64^ This effect is primarily due to the salting-out effect of salt, which enhances micelle formation by dehydrating the micellar core. This removes water and expands the micellar core by creating more room for encapsulating drug molecules.^65,66^ Among all systems, the Gelucire® 48/16:T1304 (5 : 5) formulation exhibited significantly higher %EE compared to the 3 : 7 and 7 : 3 formulations at corresponding salt concentrations, confirming the synergistic effect of balanced amphiphile composition on drug encapsulation. This statistically significant enhancement indicates a favorable contribution of balanced amphiphile composition to drug encapsulation and provides a quantitative basis for selecting the B1 formulation for subsequent biological evaluations.

Overall, the enhanced encapsulation efficiency and drug loading demonstrated in these mixed micellar systems underscore their potential for improving the solubility of QCT. By tuning micelle composition and ionic strength, these formulations can be optimized for targeted and efficient drug delivery, offering promising applications in the pharmaceutical and healthcare industries.

### 3.4. *In vitro* cell viability study

**Fig. 5:**
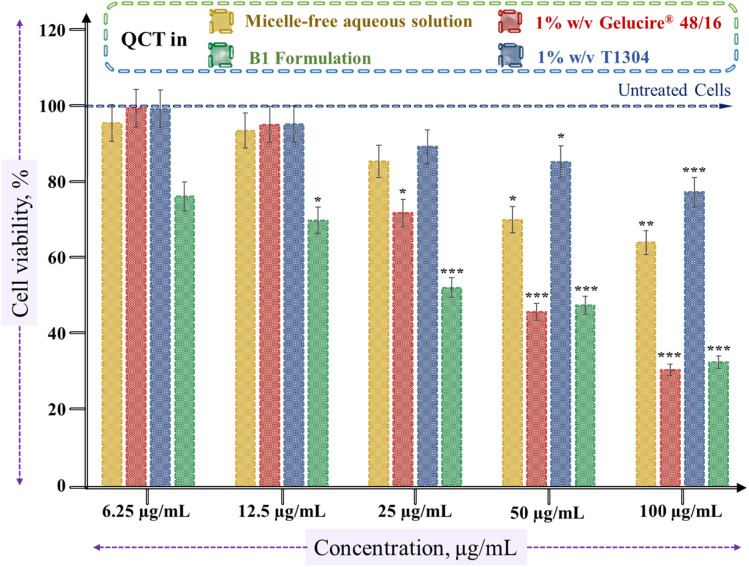
*In vitro* cell viability assay on the A549 human lung cancer cell line for the micelle-free aqueous and micellar forms of QCT. (Highly significant ***p < 0.001, very significant **p < 0.01, and significant *p < 0.05).

The cell viability of the anticancer drug against A549 lung cancer cells was evaluated in micelle-free aqueous media and after its encapsulation in different micellar systems, such as Gelucire®, T1304, and the B1 formulation, across a concentration range of 6.25–100 µg mL^−1^, as shown in Fig. 5. The results showed a concentration-dependent decrease in cell viability for all formulations, indicating that increasing drug concentrations enhanced cytotoxic effects.

Cell viability remained relatively higher across the tested concentrations when the drug was evaluated in the micelle-free aqueous media, reaching approximately 65% at a concentration of 100 µg mL^−1^. The IC_50_ value of micelle-free QCT was found to be 129.67 µg mL^−1^. Encapsulation in 1% w/v Gelucire® micelles significantly enhanced the cytotoxic effect, with a marked reduction in cell viability observed from at least 25 µg mL^−1^. At the highest concentration, Gelucire®-treated cells exhibited viability below 30%, indicating a strong therapeutic effect. This is supported by the calculated IC_50_ value of 63.51 µg mL^−1^, the lowest among all tested formulations. In contrast, the 1% w/v T1304-based micellar system demonstrated higher cell viability across all concentrations compared to Gelucire®, with an IC_50_ value of 219.77 µg mL^−1^, suggesting slower drug release. The B1 formulation exhibited cytotoxicity greater than that of T1304 alone and nearly similar to that of Gelucire®, as displayed in Fig. 5. Its IC_50_ value was determined to be 51.75 µg mL^−1^, indicating moderate potency and possible modulation in drug release kinetics due to the combination of both PEG-based nonionic surfactants.

The differences in cytotoxic performance among the micellar systems can be attributed to the interactions between the drug and the micellar core. Drug release kinetics are closely linked to a strong interaction between the hydrophobic segment and the encapsulated drug.^67^ Gelucire® has a favorable drug-binding capacity and likely supports a faster and more efficient drug release, resulting in enhanced cytotoxicity.^68^ Conversely, T1304 tends to exhibit more sustained and delayed drug release.^25^ This is consistent with findings reported by Pillai *et al.*,^18^ who observed similar sustained-release behavior of curcumin from T1304 micelles. The authors suggested that such differences in cytotoxic outcomes could be due to variations in the intracellular drug release rate from the micellar core. In another study,^25^ they checked the cytotoxicity effects of micelle-free drugs and drug-loaded T1307 micelles on cancer cells and observed that the IC_50_ values were higher for micelle-loaded drugs compared to micelle-free drugs, indicating a more sustained release from the micellar core. This controlled and prolonged release profile, although beneficial for extended drug availability, may lead to reduced immediate cytotoxicity compared to more rapidly releasing systems, like Gelucire®.

### 3.5. Morphological evaluation of A549 cells by light microscopy

**Fig. 6:**
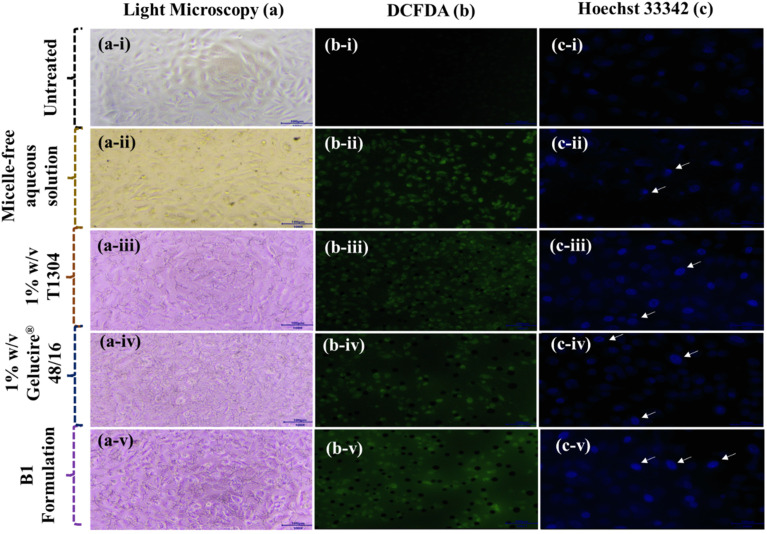
Representative images of A549 cells after treatment with different formulations. Panels show (a) light microscopy, (b) DCF fluorescence images indicating intracellular ROS levels, and (c) Hoechst staining to visualize nuclear morphology. Rows (i–v) in (a), (b) and (c) correspond to (i) untreated control cells, (ii) cells treated with a micelle-free aqueous solution, (iii) a 1% w/v T1304 micellar solution, (iv) a 1% w/v Gelucire® 48/16 micellar solution, and (v) the B1 formulation of QCT.

The morphological changes in A549 cells after treatment with QCT, 1% w/v Gelucire® 48/16, 1% w/v T1304, and their combination were examined under a light microscope, as shown in Fig. 6. In the untreated control group, cells appeared elongated, spindle-shaped, and well attached, exhibiting typical healthy morphology and uniform monolayer formation. Cells treated with a micelle-free aqueous solution of QCT showed significant morphological alterations, including cell shrinkage and loss of adherence, indicating cytotoxicity.^69^ Treatment of QCT with 1% w/v Gelucire® 48/16 led to mild alterations, with some cells displaying partial rounding and detachment, while many retained their normal morphology, suggesting moderate cytotoxic effects. In the 1% w/v T1304 micellar system, cells exhibited more noticeable morphological disruptions, such as increased rounding and disorganization, indicating greater cytotoxic impact compared to Gelucire® 48/16 alone. The apparent discrepancy between cell viability and morphological observations may be attributed to the fact that these assays reflect different cellular endpoints. Although Gelucire® 48/16 may reduce metabolic activity and viability without inducing pronounced early morphological changes, T1304 appears to interact more strongly with cell membranes and cytoskeletal organization, leading to visible cell rounding and disorganization. Thus, morphological alterations and viability loss do not necessarily occur in parallel and should be interpreted as complementary indicators of cytotoxicity. Interestingly, cells exposed to the mixed micellar formulation of B1 showed a combined morphology. Although a large portion of cells maintained attachment and shape, a subset displayed aberrant morphology, including swelling and loss of structure, suggesting moderate to significant stress on cellular integrity.^70^ These findings are directly applicable to the design of more effective and targeted anticancer therapies.

### 3.6. ROS measurement using the DCF-DA method

**Fig. 7:**
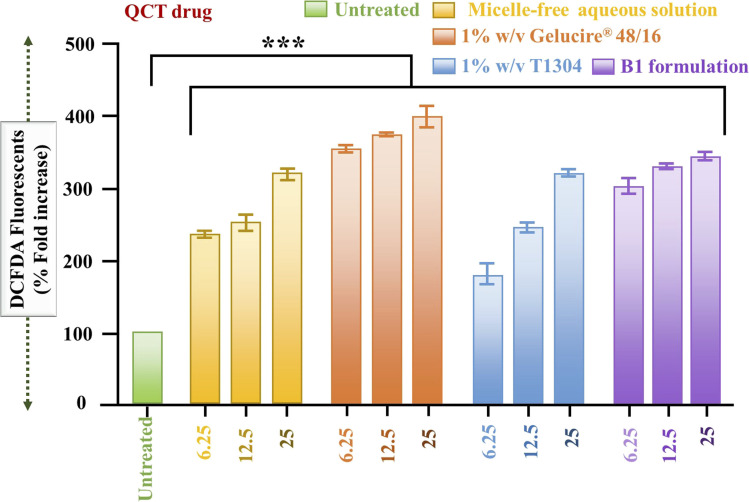
Intracellular reactive oxygen species (ROS) generation assessed *via* DCFDA fluorescence in cells treated with various formulations and concentrations of QCT. Cells were exposed to an untreated control (green), a micelle-free aqueous solution of QCT (yellow), QCT with 1% w/v Gelucire® 48/16 (orange), QCT with 1% w/v T1304 (blue), and the B1 formulation (purple) at concentrations of 6.25, 12.5, and 25 µg mL^−1^. (Highly significant: ***p < 0.001).

The DCF-DA method allows for the measurement of intracellular ROS levels, which is crucial in pharmaceutical research for evaluating oxidative stress-mediated cytotoxicity. In this study, increased ROS generation by various QCT formulations indicates their potential to induce cancer cell death, highlighting their therapeutic relevance in oncology. Fig. 6(b) displays the fluorescence microscopy images and quantitative analysis of intracellular reactive oxygen species (ROS) levels in A549 lung cancer cells treated with micelle-free aqueous solution, Gelucire® 48/16, T1304, and B1 formulation of QCT. ROS generation was assessed using the DCFH-DA assay. Treatments were administered at three concentrations: 6.25 µg, 12.5 µg, and 25 µg. The fluorescence images (Fig. 6, panel b-i to b-v) demonstrate a clear increase in intracellular ROS levels upon treatment, as evidenced by the enhanced green fluorescence intensity compared to the untreated control (b-i). Quantitative analysis confirms a statistically significant increase in ROS levels (*p* < 0.001) for all treatment groups compared to the untreated control. Among the various treatments, 1% w/v Gelucire® 48/16 (orange bars) consistently induced the highest ROS production across all concentrations, followed by the B1 formulation (purple bars), micelle-free aqueous media (yellow bars), and 1% w/v T1304 (blue bars), as shown in Fig. 7. Notably, 1% w/v T1304 at 6.25 µg exhibited the lowest ROS induction among all tested systems, indicating a comparatively milder oxidative stress response at lower doses.

These findings suggest that all micellar formulations are capable of elevating oxidative stress within cancer cells, potentially contributing to cytotoxicity. Elevated intracellular ROS levels are known to disrupt cellular homeostasis by damaging proteins, nucleic acids, lipids, and organelles, ultimately triggering apoptosis or other forms of cell death. This oxidative damage is particularly detrimental to cancer cells, which often exist near their threshold of ROS tolerance due to their high metabolic activity.^71^ Therefore, the pronounced ROS generation observed with Gelucire® 48/16 and its combination with T1304 may contribute to their therapeutic efficacy in promoting cancer cell death.

### 3.7. Hoechst 33342 staining assay

The nuclear morphology of A549 cells treated with test samples QCT in micelle-free aqueous media, Gelucire® 48/16, and T1304 micellar solution, and B1 formulation was evaluated using Hoechst 33342 staining. The untreated control group showed uniformly round and faintly stained nuclei, indicating healthy, viable cells, as shown in Fig. 6(c-i). In contrast, the cells treated with the test samples exhibited noticeable changes in nuclear morphology. Specifically, QCT in micelle-free aqueous media, Gelucire® 48/16, and T1304 micellar solution, as well as B1 formulation, showed a number of cells with condensed and brightly stained nuclei, a hallmark of DNA fragmentation and apoptotic cell death. The white arrows highlight nuclei that are fragmented DNA and condensed nuclei, as shown in Fig. 6(c-ii and c-v). This pattern reflects a combination of programmed and non-programmed cell death pathways possibly due to increased cytotoxic stress at higher or combined concentrations.^72^

Overall, Hoechst 33342 staining confirmed that the test samples induced varying degrees of DNA damage in A549 cells, with samples QCT, Gelucire® 48/16, T1304, and B1 formulation favoring apoptotic response. Such nuclear morphology analyses are critical in pharmaceutical research for evaluating the safety and efficacy of anticancer or therapeutic compounds. These findings can guide the development of formulations with optimized cell-targeting and minimal off-target toxicity.

## 4. Conclusion

This study successfully developed and characterized PEG-based mixed micellar systems using the pharmaceutical excipients, Gelucire® 48/16 and Tetronic® 1304, to enhance the solubility, encapsulation efficiency, and anticancer efficacy of quercetin (QCT), a poorly water-soluble model drug. The mixed micelles demonstrated improved salt stability and favorable morphology, as confirmed by cloud point analysis and small-angle neutron scattering. The systems retained spherical or ellipsoidal structures, with salt promoting micellar growth and drug loading *via* the salting-out effect. High-performance liquid chromatography confirmed that increasing salt concentration significantly enhanced drug encapsulation efficiency, particularly in the balanced Gelucire® 48/16:T1304 formulation. Micellar encapsulation of QCT significantly improved its cytotoxicity against A549 lung cancer cells, with Gelucire® 48/16 showing the strongest effect (lowest IC_50_ and highest ROS generation). The B1 formulation enhanced cytotoxicity, while T1304 showed weaker activity due to slower drug release. QCT in micelle-free aqueous media, Gelucire® 48/16, and T1304 micellar solution, as well as B1 formulation, primarily induced apoptotic cell death *via* mitochondrial dysfunction, ROS generation, and DNA damage. Beyond mechanistic understanding, this study underscores the industrial and healthcare relevance of such systems. By combining high encapsulation efficiency with tunable release kinetics, mixed micelles represent a practical platform for bridging laboratory research with industrial healthcare applications, addressing the long-standing challenge of poorly soluble drug formulations. Overall, the results highlight that PEG-based mixed micellar systems support their application as promising drug delivery systems for improving the therapeutic efficacy of poorly water-soluble anticancer drugs, like quercetin. Further studies should be conducted for targeted drug delivery applications and potential anticancer effects.

## Conflicts of interest

The authors declare that they have no known competing financial interests/personal relationships that could have appeared to influence the work reported in this paper.

## Data Availability

Data will be made available on request.
